# Crystal structure of a new 2,6-bis­(imino)­pyridine derivative: (1*E*,1′*E*)-1,1′-(pyridine-2,6-di­yl)bis­[*N*-(4-chloro­phen­yl)ethan-1-imine]

**DOI:** 10.1107/S2056989018017966

**Published:** 2019-01-04

**Authors:** Rajagopal Rajesh, E. S. Sella, Olivier Blacque, Kunjanpillai Rajesh

**Affiliations:** a Department of Chemistry, St. Albert’s College (Autonomous), Ernakulam, Kochi, Kerala 682018, India; b Department of Chemistry, University of Zurich, Winterthurerstrasse 190, 8057 Zurich, Switzerland

**Keywords:** crystal structure, imino­pyridine, 2,6-bis­(imino)­pyridine derivative, C—H⋯π inter­actions

## Abstract

The asymmetric unit of the title compound, C_21_H_17_Cl_2_N_3_, contains two crystallographically independent mol­ecules (*A* and *B*). Both mol­ecules have *E* configurations for both imine double bonds with regard to the aryl and pyridine groups. The conformations of the two mol­ecules differ with the 4-chloro­phenyl rings being inclined to the central pyridine ring by 77.64 (6) and 86.18 (6)° in mol­ecule *A*, and 80.02 (5) and 43.41 (6)° in mol­ecule *B*. In the crystal, mol­ecules are linked by a number of C—H⋯π inter­actions, forming layers parallel to the *bc* plane.

## Abstract 2

This new 2,6-bis­(imino)­pyridine derivative with terminal 4-chloro­phenyl rings crystallizes with two independent mol­ecules in the asymmetric unit.

## Chemical context   

2,6-Bis(imino)­pyridines have acquired widespread inter­est because of their potential application as ligands in olefin polymerization reactions: see, for example, the work of Antonov *et al.* (2012 ▸) or Kawakami *et al.* (2015 ▸). Metal complexes of such ligands have been applied to aryl C—H activation (Dayan *et al.*, 2010 ▸; Sigen *et al.*, 2013 ▸) and transfer hydrogenation reactions (Dayan & Çetinkaya, 2007 ▸). As a result of the redox activity of the ligand (Noss *et al.*, 2018 ▸), electrochemical and luminescent properties of its complexes have been reported (Fan *et al.*, 2004 ▸). Recently, the biomimetic reactivity of Zn–alkyl complexes has also been revealed (Sandoval *et al.*, 2018 ▸). We report herein on the crystal structure of a new 2,6-bis­(imino)­pyridine derivative with terminal 4-chloro­phenyl rings.
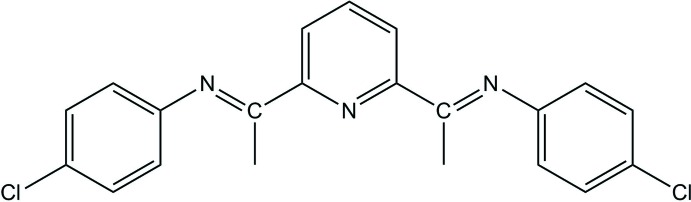


## Structural commentary   

**Figure 1:**
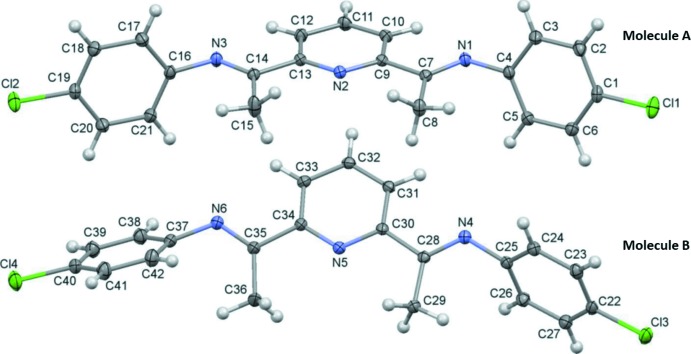
Mol­ecular structure of the title compound showing the two crystallographically independent mol­ecules (*A* and *B*), with the atom labelling. Displacement ellipsoids drawn at the 30% probability level.

The asymmetric unit of the title compound contains two crystallographically independent mol­ecules (*A* and *B*), illustrated in Fig. 1 ▸. Both mol­ecules have *E*-configurations for both imine double bonds with regard to the aryl and pyridine groups. The C=N bond lengths of the imine groups are in a narrow range, 1.2675 (15) to 1.2808 (14) Å (Table 1 ▸). These values are similar to the C=N bond lengths found in the crystal structures of other 2,6-bis­(imino)­pyridyl ligands; for example 1.266 (4) Å in the ‘parent’ compound 2,6-bis­[1-(phenyl­imino)­eth­yl]pyridine (Mentes *et al.*, 2001 ▸).

**Figure 2:**
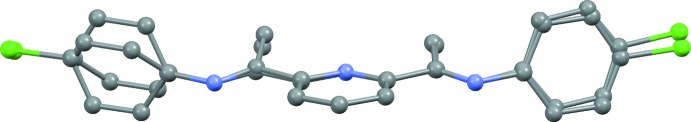
View of the mol­ecular overlay of the two independent mol­ecules.

In mol­ecule *A*, the 4-chloro­phenyl rings (C1–C6 and C16–C21) are inclined to the central pyridine ring (N2/C9–C13) by 77.64 (6) and 86.18 (6)°, respectively. In mol­ecule *B*, the dihedral angles between the 4-chloro­phenyl rings (C22–C27 and C37–C42) and the central pyridine ring (N5/C30–C34) are 80.02 (5) and 43.41 (6)°, respectively. The terminal ring (C37–C42) in mol­ecule *B* adopts a significantly different conformation from the other benzene rings, as shown in Fig. 2 ▸, a mol­ecular overlay figure calculated with *Mercury* (Macrae *et al.*, 2008 ▸).

## Supra­molecular features   

**Figure 3:**
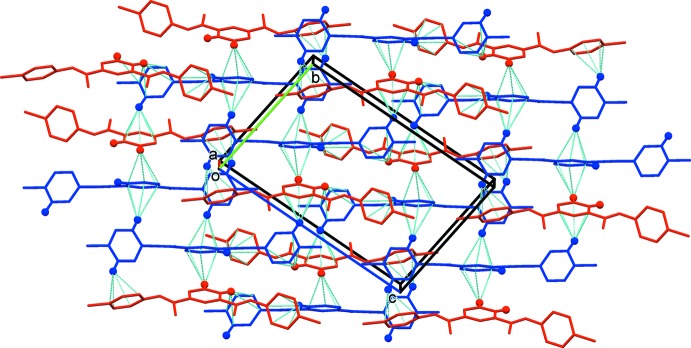
A view along the *a* axis of the crystal packing of the title compound, showing the C—H⋯π inter­actions as dashed lines (Table 2 ▸; colour code: mol­ecule *A* blue, mol­ecule *B* red). Only the H atoms (blue and red balls) involved in these inter­actions have been included.

In the crystal, mol­ecules are linked by a series of C—H⋯π inter­actions, forming layers lying parallel to the *bc* plane (Table 2 ▸ and Fig. 3 ▸). There are no other significant inter­molecular inter­actions present in the crystal structure. All H⋯N and H⋯Cl inter­molecular distances exceed the sum of their van der Waals radii.

## Database survey   

A search of the Cambridge Structural Database (CSD, V5.39, last update August 2018; Groom *et al.*, 2016 ▸) confirmed that 2,6-bis­(imino)­pyridine derivatives are widely used as trident­ate chelating ligands for transition metals (more than 600 hits). A search for the substructure 1,1′-(pyridine-2,6-di­yl)bis­(*N*-(phen­yl)ethan-1-imine) gave 25 hits. The crystal structure of the 2,6-bis­[1-(phenyl­imino)­eth­yl]pyridine mol­ecule was reported in 2001 (CSD refcode QOQROD; Mentes *et al.*, 2001 ▸). The first crystal structure with that mol­ecule used a tridentate ligand for a transition metal (*M* = Ni) was reported earlier in 1975 (PIEPNI10; Alyea *et al.*, 1975 ▸). The crystal structure of the bis­(4-meth­oxy­phen­yl) derivative has also been reported (REMSEH; Meehan *et al.*, 1997 ▸). In the 25 structures deposited in the CSD, the C=N bond lengths range from *ca* 1.262–1.294 Å and the dihedral angles involving the outer benzene rings with respect to the central pyridine ring range from *ca* 52.75 to 88.76°. In QOQROD and REMSEH, which both possess mirror symmetry, the C=N bond lengths are 1.266 (4) and 1.274 (5) Å, respectively, while the benzene rings are inclined to the central pyridine ring by 60.2 (2) and 55.2 (2)°, respectively. While the conformation of mol­ecule *A* conforms to the overall limits, that of mol­ecule *B* does not, with the terminal ring (C37–C42) being inclined to the pyridine ring by only 43.41 (6)°.

The crystal structures of two 2,6-dihalogeno (*X* = Cl, Br) derivatives have also been reported, *viz*. 2,6-bis­[1-(2,6-di­bromo­phenyl­imino)­eth­yl]pyridine (EMEJIP; Chen *et al.*, 2003 ▸) and 2,6-bis­[1-(2,6-di­chloro­phenyl­imino)­eth­yl]pyridine (EYACUD; Sieh *et al.*, 2011 ▸). Both compounds have *E* configurations around both C=N imine bonds. Owing to steric hindrance, the 2,6-dihalophenyl rings are inclined to the central pyridine ring by 85.7 (3) and 88.0 (3)° in EMEJIP and 81.13 (6) and 74.22 (7)° in EYACUD. In the crystals of these two compounds, as in the crystal of the title compound, the H⋯N and H⋯Br/Cl inter­molecular distances all exceed the sum of their van der Waals radii.

## Synthesis and crystallization   

To a solution of 2,6-di­acetyl­pyridine (0.5 g, 3.06 mmol) and *p*-chloro­aniline (0.977 g, 7.66 mmol) in 20 ml of toluene was added 20 mg of *p*-toluene­sulfonic acid (Görl *et al.*, 2011 ▸). The reaction mixture was refluxed for 24 h using a Dean–Stark trap, then cooled to room temperature and 50 ml of saturated sodium bicarbonate solution was added. The organic layer was separated and filtered over sodium sulfate. The solvent was removed in a rotary evaporator giving a light-brown-coloured mass. Ethanol (*ca* 25 ml) was added to this solid mass followed by the addition of hexane (*ca* 10 ml). The solution was then kept in the deep-freezer at 253 K. The title compound was obtained as a yellow solid in 31% yield (0.363 g, 0.95 mmol). A very dilute solution of the compound was prepared in a 1:1 mixture of ethanol and hexane. On slow evaporation of the solvents at room temperature, pale-yellow crystals were obtained over a period of two weeks.

An alternate method for the synthesis is as follows: To a solution of 2,6-di­acetyl­pyridine (0.5 g, 3.06 mmol) and *p*-chloro­aniline (0.782 g, 6.13 mmol) in 5 mL of absolute ethanol was added three drops of acetic acid. The reaction mixture was refluxed for 24 h, cooled to room temperature and then approximately 15 mL of hexane were added. The mixture was heated on a water bath and filtered hot using filter paper. The solution was kept in a deep freezer at 253 K. The title compound was obtained as a yellow solid in 26% yield (0.305 g, 0.80 mmol).

*Spectroscopic data:* IR (ATR, cm^−1^): 3072 (*w*), 1638 (*s*), 1567 (*w*), 1482 (*s*), 1450 (*w*), 1362 (*s*), 1322 (*w*), 1297 (*m*), 1216 (*s*), 1171 (*w*), 1148 (*w*), 1119 (*m*), 1091 (*m*), 1010 (*w*), 994 (*w*), 955 (*w*), 842 (*s*), 787 (*s*), 743 (*w*), 723 (*m*), 672 (*m*), 635 (*w*), 597 (*m*), 532 (*w*), 517 (*m*); ^1^H NMR (400 MHz, CDCl_3_): 2.40 (*s*, 6H), 6.79 (*d*, *J* = 8.5 Hz, 4H), 7.35 (*d*, *J* = 8.5 Hz, 4H), 7.88 (*t*, *J* = 7.8 Hz, 1H), 8.32 (*d*, *J* = 7.8 Hz, 2H); ^13^C NMR (75 MHz, CDCl_3_,): 16.6, 121.0, 122.9, 129.4, 129.5, 137.3, 150.0, 155.6, 168.3.

## Refinement   

Crystal data, data collection and structure refinement details are summarized in Table 3 ▸. All H atoms were placed in calculated positions and refined as riding atoms: C—H = 0.95–0.98 Å with *U*_iso_(H) = 1.5*U*_eq_(C-meth­yl) and 1.2*U*_eq_(C) for other H atoms.

## Supplementary Material

Crystal structure: contains datablock(s) Global, I. DOI: 10.1107/S2056989018017966/su5463sup1.cif

Structure factors: contains datablock(s) I. DOI: 10.1107/S2056989018017966/su5463Isup2.hkl

CCDC reference: 1886124

Additional supporting information:  crystallographic information; 3D view; checkCIF report

## Figures and Tables

**Table 1:** Selected bond lengths (Å)

C7—N1	1.2772 (14)	C28—N4	1.2808 (14)
C14—N3	1.2696 (14)	C35—N6	1.2675 (15)

**Table 2:** Hydrogen-bond geometry (Å, °) *Cg*1, *Cg*2, *Cg*4, *Cg*5 and *Cg*6 are the centroids of rings N2/C9–C13, C1–C6, N5/C30–C34, C22–C27 and C37–C42, respectively.

*D*—H⋯*A*	*D*—H	H⋯*A*	*D*⋯*A*	*D*—H⋯*A*
C20—H20⋯*Cg*6	0.95	2.94	3.6735 (14)	135
C32—H32⋯*Cg*1	0.95	2.73	3.3273 (12)	121
C2—H2⋯*Cg*4^i^	0.95	2.67	3.4012 (13)	134
C10—H10⋯*Cg*5^ii^	0.95	2.81	3.6446 (13)	147
C17—H17⋯*Cg*1^iii^	0.95	2.70	3.5850 (14)	155
C31—H31⋯*Cg*2^ii^	0.95	2.93	3.5795 (12)	127

Symmetry codes: (i) 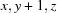; (ii) 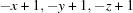; (iii) 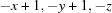.

**Table 3:** Experimental details

Crystal data	
Chemical formula	C_21_H_17_Cl_2_N_3_
*M*_r_	382.27
Crystal system, space group	Triclinic, *P* 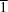
Temperature (K)	160
*a*, *b*, *c* (Å)	10.5375 (2), 10.8479 (2), 16.8936 (3)
α, β, γ (°)	82.261 (2), 88.543 (1), 84.930 (2)
*V* (Å^3^)	1905.85 (6)
*Z*	4
Radiation type	Mo *K*α
μ (mm^−1^)	0.35
Crystal size (mm)	0.36 × 0.28 × 0.20

Data collection	
Diffractometer	XtaLAB Synergy, Dualflex, Pilatus 200K
Absorption correction	Analytical (*CrysAlis PRO*; Rigaku OD, 2018 ▸)
*T*_min_, *T*_max_	0.919, 0.941
No. of measured, independent and observed [*I* > 2σ(*I*)] reflections	54775, 11604, 9934
*R*_int_	0.027
(sin θ/λ)_max_ (Å^−1^)	0.714

Refinement	
*R*[*F* ^2^ > 2σ(*F* ^2^)], *wR*(*F* ^2^), *S*	0.039, 0.111, 1.06
No. of reflections	11604
No. of parameters	473
H-atom treatment	H-atom parameters constrained
Δρ_max_, Δρ_min_ (e Å^−3^)	0.47, −0.67

Computer programs: *CrysAlis PRO* (Rigaku OD, 2018 ▸), *SHELXT* (Sheldrick, 2015*a* ▸), *SHELXL2018* (Sheldrick, 2015*b* ▸), *OLEX2* (Dolomanov *et al.*, 2009 ▸) and *Mercury* (Macrae *et al.*, 2008 ▸).

## supplementary crystallographic information

### Crystal data

**Table:**

C_21_H_17_Cl_2_N_3_	*Z* = 4
*M**_r_* = 382.27	*F*(000) = 792
Triclinic, *P*1	*D*_x_ = 1.332 Mg m^−^^3^
*a* = 10.5375 (2) Å	Mo *K*α radiation, λ = 0.71073 Å
*b* = 10.8479 (2) Å	Cell parameters from 27060 reflections
*c* = 16.8936 (3) Å	θ = 2.3–33.2°
α = 82.261 (2)°	µ = 0.35 mm^−^^1^
β = 88.543 (1)°	*T* = 160 K
γ = 84.930 (2)°	Block, pale yellow
*V* = 1905.85 (6) Å^3^	0.36 × 0.28 × 0.20 mm

### Data collection

**Table:**

XtaLAB Synergy, Dualflex, Pilatus 200K diffractometer	11604 independent reflections
Radiation source: micro-focus sealed X-ray tube, PhotonJet (Mo) X-ray Source	9934 reflections with *I* > 2σ(*I*)
Mirror monochromator	*R*_int_ = 0.027
ω scans	θ_max_ = 30.5°, θ_min_ = 2.1°
Absorption correction: analytical (CrysAlis PRO; Rigaku OD, 2018)	*h* = −14→15
*T*_min_ = 0.919, *T*_max_ = 0.941	*k* = −15→15
54775 measured reflections	*l* = −24→24

### Refinement

**Table:**

Refinement on *F*^2^	Primary atom site location: dual
Least-squares matrix: full	Secondary atom site location: difference Fourier map
*R*[*F*^2^ > 2σ(*F*^2^)] = 0.039	Hydrogen site location: inferred from neighbouring sites
*wR*(*F*^2^) = 0.111	H-atom parameters constrained
*S* = 1.06	*w* = 1/[σ^2^(*F*_o_^2^) + (0.0577*P*)^2^ + 0.5294*P*] where *P* = (*F*_o_^2^ + 2*F*_c_^2^)/3
11604 reflections	(Δ/σ)_max_ = 0.001
473 parameters	Δρ_max_ = 0.47 e Å^−^^3^
0 restraints	Δρ_min_ = −0.67 e Å^−^^3^

### Special details

**Table:**

Geometry. All esds (except the esd in the dihedral angle between two l.s. planes) are estimated using the full covariance matrix. The cell esds are taken into account individually in the estimation of esds in distances, angles and torsion angles; correlations between esds in cell parameters are only used when they are defined by crystal symmetry. An approximate (isotropic) treatment of cell esds is used for estimating esds involving l.s. planes.

### Fractional atomic coordinates and isotropic or equivalent isotropic displacement parameters (Å^2^)

**Table:**

	*x*	*y*	*z*	*U*_iso_*/*U*_eq_	
C1	0.33253 (11)	0.84351 (11)	0.52377 (7)	0.0277 (2)	
C2	0.39558 (13)	0.89874 (11)	0.45751 (7)	0.0319 (2)	
H2	0.403602	0.986096	0.450265	0.038*	
C3	0.44713 (13)	0.82499 (11)	0.40155 (7)	0.0310 (2)	
H3	0.492617	0.861820	0.356481	0.037*	
C4	0.43261 (11)	0.69734 (10)	0.41106 (7)	0.0254 (2)	
C5	0.37049 (12)	0.64286 (11)	0.47914 (7)	0.0282 (2)	
H5	0.362343	0.555521	0.486755	0.034*	
C6	0.32057 (11)	0.71611 (11)	0.53576 (7)	0.0285 (2)	
H6	0.278606	0.679231	0.582298	0.034*	
C7	0.42305 (10)	0.57357 (10)	0.30817 (6)	0.02233 (19)	
C8	0.27998 (11)	0.58318 (13)	0.30540 (8)	0.0320 (2)	
H8A	0.248873	0.504655	0.330749	0.048*	
H8B	0.252739	0.599462	0.249660	0.048*	
H8C	0.245071	0.651695	0.333999	0.048*	
C9	0.49567 (10)	0.49648 (9)	0.25206 (6)	0.02101 (19)	
C10	0.62744 (11)	0.47078 (10)	0.25912 (7)	0.0249 (2)	
H10	0.672537	0.503405	0.298482	0.030*	
C11	0.69096 (11)	0.39668 (12)	0.20746 (7)	0.0291 (2)	
H11	0.780494	0.377219	0.211023	0.035*	
C12	0.62190 (11)	0.35128 (11)	0.15043 (7)	0.0271 (2)	
H12	0.663229	0.300253	0.114345	0.032*	
C13	0.49066 (10)	0.38206 (10)	0.14718 (6)	0.02198 (19)	
C14	0.41233 (10)	0.33185 (10)	0.08780 (6)	0.0232 (2)	
C15	0.27004 (12)	0.35106 (16)	0.09431 (10)	0.0425 (3)	
H15A	0.231832	0.322186	0.048576	0.064*	
H15B	0.244269	0.440024	0.094688	0.064*	
H15C	0.240977	0.303534	0.143916	0.064*	
C16	0.41268 (10)	0.21412 (11)	−0.01944 (7)	0.0252 (2)	
C17	0.41070 (12)	0.26750 (12)	−0.09902 (7)	0.0306 (2)	
H17	0.438136	0.348713	−0.113385	0.037*	
C18	0.36890 (12)	0.20299 (12)	−0.15767 (7)	0.0304 (2)	
H18	0.367791	0.239417	−0.212058	0.036*	
C19	0.32891 (11)	0.08498 (11)	−0.13579 (7)	0.0267 (2)	
C20	0.32610 (13)	0.03190 (12)	−0.05683 (7)	0.0320 (2)	
H20	0.295806	−0.048126	−0.042522	0.038*	
C21	0.36821 (13)	0.09712 (12)	0.00144 (7)	0.0322 (2)	
H21	0.366580	0.061451	0.055952	0.039*	
Cl1	0.26945 (4)	0.93500 (3)	0.59507 (2)	0.04153 (9)	
Cl2	0.28305 (4)	0.00035 (3)	−0.20954 (2)	0.04044 (9)	
N1	0.49029 (10)	0.62550 (10)	0.35388 (6)	0.0281 (2)	
N2	0.42787 (9)	0.45348 (8)	0.19705 (5)	0.02178 (17)	
N3	0.47403 (10)	0.27235 (10)	0.03734 (6)	0.0296 (2)	
C22	0.04883 (11)	0.44751 (12)	0.72766 (7)	0.0278 (2)	
C23	0.04388 (11)	0.53195 (11)	0.65837 (8)	0.0293 (2)	
H23	0.005799	0.614549	0.658940	0.035*	
C24	0.09527 (11)	0.49444 (11)	0.58799 (7)	0.0267 (2)	
H24	0.093327	0.552097	0.540346	0.032*	
C25	0.14964 (10)	0.37279 (10)	0.58684 (7)	0.0238 (2)	
C26	0.15381 (12)	0.28959 (11)	0.65750 (7)	0.0298 (2)	
H26	0.191543	0.206783	0.657329	0.036*	
C27	0.10335 (12)	0.32680 (12)	0.72790 (7)	0.0305 (2)	
H27	0.106202	0.269881	0.775889	0.037*	
C28	0.16246 (10)	0.25644 (10)	0.47943 (6)	0.02186 (19)	
C29	0.04079 (11)	0.19559 (11)	0.50021 (7)	0.0266 (2)	
H29A	0.060896	0.106970	0.519780	0.040*	
H29B	−0.011964	0.203652	0.452537	0.040*	
H29C	−0.005754	0.236549	0.541826	0.040*	
C30	0.23750 (10)	0.21730 (10)	0.40933 (6)	0.02175 (19)	
C31	0.36130 (10)	0.25147 (10)	0.39370 (7)	0.0236 (2)	
H31	0.399260	0.302265	0.426474	0.028*	
C32	0.42741 (11)	0.20937 (10)	0.32913 (7)	0.0250 (2)	
H32	0.511683	0.231219	0.316838	0.030*	
C33	0.36935 (11)	0.13513 (10)	0.28271 (7)	0.0253 (2)	
H33	0.412512	0.106145	0.237813	0.030*	
C34	0.24608 (11)	0.10387 (10)	0.30336 (6)	0.0244 (2)	
C35	0.18171 (12)	0.01753 (12)	0.25878 (7)	0.0290 (2)	
C36	0.0712 (2)	−0.0429 (2)	0.29993 (11)	0.0687 (7)	
H36A	−0.003006	0.018417	0.298850	0.103*	
H36B	0.093131	−0.074184	0.355476	0.103*	
H36C	0.051163	−0.112627	0.272397	0.103*	
C37	0.18205 (12)	−0.08525 (11)	0.14453 (7)	0.0274 (2)	
C38	0.26630 (12)	−0.18181 (13)	0.12313 (8)	0.0332 (3)	
H38	0.349787	−0.193775	0.144341	0.040*	
C39	0.23006 (13)	−0.26095 (13)	0.07117 (8)	0.0351 (3)	
H39	0.287777	−0.327254	0.057339	0.042*	
C40	0.10945 (13)	−0.24197 (12)	0.04003 (7)	0.0321 (2)	
C41	0.02384 (13)	−0.14670 (14)	0.06006 (8)	0.0377 (3)	
H41	−0.059284	−0.135010	0.038303	0.045*	
C42	0.06052 (13)	−0.06793 (13)	0.11246 (8)	0.0355 (3)	
H42	0.002237	−0.002089	0.126326	0.043*	
Cl3	−0.01235 (3)	0.49452 (4)	0.81635 (2)	0.04202 (9)	
Cl4	0.06545 (4)	−0.33813 (4)	−0.02725 (2)	0.04994 (10)	
N4	0.20804 (9)	0.33848 (9)	0.51589 (6)	0.02557 (18)	
N5	0.18092 (9)	0.14407 (9)	0.36555 (6)	0.02414 (18)	
N6	0.23082 (10)	−0.00296 (10)	0.19202 (6)	0.0294 (2)	

### Atomic displacement parameters (Å^2^)

**Table:**

	*U*^11^	*U*^22^	*U*^33^	*U*^12^	*U*^13^	*U*^23^
C1	0.0296 (5)	0.0297 (5)	0.0252 (5)	0.0036 (4)	−0.0053 (4)	−0.0117 (4)
C2	0.0443 (7)	0.0233 (5)	0.0289 (6)	−0.0020 (5)	−0.0045 (5)	−0.0058 (4)
C3	0.0413 (7)	0.0274 (5)	0.0253 (5)	−0.0070 (5)	0.0014 (5)	−0.0050 (4)
C4	0.0256 (5)	0.0270 (5)	0.0259 (5)	−0.0043 (4)	−0.0019 (4)	−0.0107 (4)
C5	0.0313 (6)	0.0251 (5)	0.0301 (5)	−0.0071 (4)	0.0012 (4)	−0.0081 (4)
C6	0.0292 (5)	0.0325 (6)	0.0251 (5)	−0.0054 (4)	0.0014 (4)	−0.0068 (4)
C7	0.0262 (5)	0.0204 (4)	0.0209 (5)	−0.0038 (4)	0.0006 (4)	−0.0036 (4)
C8	0.0251 (5)	0.0400 (6)	0.0336 (6)	0.0012 (5)	−0.0030 (4)	−0.0163 (5)
C9	0.0240 (5)	0.0193 (4)	0.0203 (4)	−0.0050 (4)	0.0009 (4)	−0.0031 (3)
C10	0.0239 (5)	0.0269 (5)	0.0256 (5)	−0.0068 (4)	−0.0011 (4)	−0.0062 (4)
C11	0.0206 (5)	0.0355 (6)	0.0337 (6)	−0.0050 (4)	0.0003 (4)	−0.0113 (5)
C12	0.0232 (5)	0.0316 (5)	0.0284 (5)	−0.0040 (4)	0.0028 (4)	−0.0108 (4)
C13	0.0238 (5)	0.0219 (4)	0.0213 (5)	−0.0053 (4)	0.0013 (4)	−0.0049 (4)
C14	0.0229 (5)	0.0244 (5)	0.0231 (5)	−0.0032 (4)	−0.0008 (4)	−0.0059 (4)
C15	0.0246 (6)	0.0600 (9)	0.0490 (8)	0.0030 (6)	−0.0048 (5)	−0.0327 (7)
C16	0.0210 (5)	0.0317 (5)	0.0251 (5)	−0.0023 (4)	0.0021 (4)	−0.0118 (4)
C17	0.0344 (6)	0.0298 (5)	0.0289 (6)	−0.0079 (5)	−0.0029 (5)	−0.0048 (4)
C18	0.0337 (6)	0.0351 (6)	0.0228 (5)	−0.0060 (5)	−0.0031 (4)	−0.0032 (4)
C19	0.0287 (5)	0.0293 (5)	0.0237 (5)	−0.0006 (4)	−0.0033 (4)	−0.0102 (4)
C20	0.0405 (7)	0.0296 (6)	0.0275 (6)	−0.0097 (5)	−0.0028 (5)	−0.0048 (4)
C21	0.0381 (6)	0.0394 (6)	0.0214 (5)	−0.0125 (5)	0.0014 (4)	−0.0060 (4)
Cl1	0.04879 (19)	0.04263 (17)	0.03431 (16)	0.01175 (14)	−0.00271 (13)	−0.01911 (13)
Cl2	0.0540 (2)	0.03864 (16)	0.03227 (16)	−0.00355 (14)	−0.01178 (13)	−0.01599 (12)
N1	0.0285 (5)	0.0300 (5)	0.0288 (5)	−0.0063 (4)	0.0019 (4)	−0.0130 (4)
N2	0.0237 (4)	0.0214 (4)	0.0212 (4)	−0.0038 (3)	0.0001 (3)	−0.0049 (3)
N3	0.0253 (5)	0.0389 (5)	0.0282 (5)	−0.0061 (4)	0.0019 (4)	−0.0157 (4)
C22	0.0203 (5)	0.0394 (6)	0.0273 (5)	−0.0086 (4)	0.0029 (4)	−0.0144 (4)
C23	0.0247 (5)	0.0302 (5)	0.0349 (6)	−0.0012 (4)	−0.0006 (4)	−0.0121 (5)
C24	0.0276 (5)	0.0268 (5)	0.0264 (5)	−0.0031 (4)	−0.0023 (4)	−0.0056 (4)
C25	0.0232 (5)	0.0258 (5)	0.0242 (5)	−0.0057 (4)	0.0000 (4)	−0.0074 (4)
C26	0.0363 (6)	0.0255 (5)	0.0277 (5)	−0.0021 (4)	0.0000 (5)	−0.0048 (4)
C27	0.0336 (6)	0.0344 (6)	0.0246 (5)	−0.0089 (5)	0.0003 (4)	−0.0045 (4)
C28	0.0237 (5)	0.0209 (4)	0.0208 (4)	−0.0013 (4)	−0.0001 (4)	−0.0024 (3)
C29	0.0263 (5)	0.0282 (5)	0.0267 (5)	−0.0058 (4)	0.0039 (4)	−0.0068 (4)
C30	0.0246 (5)	0.0204 (4)	0.0204 (4)	−0.0035 (4)	0.0006 (4)	−0.0026 (3)
C31	0.0251 (5)	0.0216 (4)	0.0246 (5)	−0.0054 (4)	−0.0011 (4)	−0.0025 (4)
C32	0.0241 (5)	0.0246 (5)	0.0261 (5)	−0.0067 (4)	0.0021 (4)	−0.0005 (4)
C33	0.0280 (5)	0.0265 (5)	0.0217 (5)	−0.0057 (4)	0.0047 (4)	−0.0028 (4)
C34	0.0289 (5)	0.0256 (5)	0.0200 (5)	−0.0079 (4)	0.0025 (4)	−0.0045 (4)
C35	0.0331 (6)	0.0335 (6)	0.0233 (5)	−0.0130 (5)	0.0051 (4)	−0.0082 (4)
C36	0.0795 (13)	0.1008 (15)	0.0441 (9)	−0.0696 (12)	0.0337 (9)	−0.0417 (9)
C37	0.0318 (6)	0.0315 (5)	0.0207 (5)	−0.0096 (4)	0.0043 (4)	−0.0068 (4)
C38	0.0297 (6)	0.0390 (6)	0.0332 (6)	−0.0049 (5)	−0.0012 (5)	−0.0119 (5)
C39	0.0349 (6)	0.0355 (6)	0.0375 (6)	−0.0036 (5)	0.0025 (5)	−0.0145 (5)
C40	0.0371 (6)	0.0371 (6)	0.0256 (5)	−0.0127 (5)	0.0025 (5)	−0.0113 (5)
C41	0.0329 (6)	0.0483 (7)	0.0345 (6)	−0.0032 (5)	−0.0057 (5)	−0.0139 (6)
C42	0.0337 (6)	0.0416 (7)	0.0332 (6)	0.0003 (5)	−0.0008 (5)	−0.0140 (5)
Cl3	0.03482 (16)	0.0619 (2)	0.03569 (16)	−0.01407 (14)	0.01212 (12)	−0.02555 (15)
Cl4	0.0486 (2)	0.0618 (2)	0.0484 (2)	−0.01678 (17)	−0.00091 (16)	−0.03205 (18)
N4	0.0283 (5)	0.0257 (4)	0.0239 (4)	−0.0049 (4)	0.0019 (4)	−0.0067 (3)
N5	0.0257 (4)	0.0267 (4)	0.0212 (4)	−0.0071 (3)	0.0022 (3)	−0.0052 (3)
N6	0.0327 (5)	0.0343 (5)	0.0237 (4)	−0.0107 (4)	0.0043 (4)	−0.0092 (4)

### Geometric parameters (Å, º)

**Table:**

C1—C2	1.3803 (18)	C22—C23	1.3849 (18)
C1—C6	1.3860 (17)	C22—C27	1.3823 (18)
C1—Cl1	1.7442 (11)	C22—Cl3	1.7376 (12)
C2—H2	0.9500	C23—H23	0.9500
C2—C3	1.3891 (17)	C23—C24	1.3895 (16)
C3—H3	0.9500	C24—H24	0.9500
C3—C4	1.3939 (16)	C24—C25	1.3939 (16)
C4—C5	1.3956 (17)	C25—C26	1.3953 (16)
C4—N1	1.4138 (14)	C25—N4	1.4128 (14)
C5—H5	0.9500	C26—H26	0.9500
C5—C6	1.3887 (16)	C26—C27	1.3854 (17)
C6—H6	0.9500	C27—H27	0.9500
C7—C8	1.5035 (16)	C28—C29	1.5057 (15)
C7—C9	1.4989 (15)	C28—C30	1.4955 (15)
C7—N1	1.2772 (14)	C28—N4	1.2808 (14)
C8—H8A	0.9800	C29—H29A	0.9800
C8—H8B	0.9800	C29—H29B	0.9800
C8—H8C	0.9800	C29—H29C	0.9800
C9—C10	1.3968 (15)	C30—C31	1.3966 (15)
C9—N2	1.3420 (13)	C30—N5	1.3408 (13)
C10—H10	0.9500	C31—H31	0.9500
C10—C11	1.3856 (16)	C31—C32	1.3868 (16)
C11—H11	0.9500	C32—H32	0.9500
C11—C12	1.3882 (16)	C32—C33	1.3853 (15)
C12—H12	0.9500	C33—H33	0.9500
C12—C13	1.3945 (16)	C33—C34	1.3953 (15)
C13—C14	1.5009 (14)	C34—C35	1.4942 (15)
C13—N2	1.3433 (13)	C34—N5	1.3414 (14)
C14—C15	1.4987 (17)	C35—C36	1.4967 (18)
C14—N3	1.2696 (14)	C35—N6	1.2675 (15)
C15—H15A	0.9800	C36—H36A	0.9800
C15—H15B	0.9800	C36—H36B	0.9800
C15—H15C	0.9800	C36—H36C	0.9800
C16—C17	1.3902 (17)	C37—C38	1.3919 (18)
C16—C21	1.3895 (17)	C37—C42	1.3907 (18)
C16—N3	1.4156 (14)	C37—N6	1.4141 (14)
C17—H17	0.9500	C38—H38	0.9500
C17—C18	1.3888 (16)	C38—C39	1.3898 (17)
C18—H18	0.9500	C39—H39	0.9500
C18—C19	1.3831 (17)	C39—C40	1.3758 (19)
C19—C20	1.3805 (17)	C40—C41	1.382 (2)
C19—Cl2	1.7453 (11)	C40—Cl4	1.7397 (12)
C20—H20	0.9500	C41—H41	0.9500
C20—C21	1.3899 (16)	C41—C42	1.3938 (18)
C21—H21	0.9500	C42—H42	0.9500
			
C2—C1—C6	121.33 (11)	C23—C22—Cl3	119.53 (10)
C2—C1—Cl1	119.49 (9)	C27—C22—C23	121.20 (11)
C6—C1—Cl1	119.17 (9)	C27—C22—Cl3	119.25 (10)
C1—C2—H2	120.4	C22—C23—H23	120.4
C1—C2—C3	119.17 (11)	C22—C23—C24	119.22 (11)
C3—C2—H2	120.4	C24—C23—H23	120.4
C2—C3—H3	119.8	C23—C24—H24	119.8
C2—C3—C4	120.48 (11)	C23—C24—C25	120.45 (11)
C4—C3—H3	119.8	C25—C24—H24	119.8
C3—C4—C5	119.47 (10)	C24—C25—C26	119.23 (10)
C3—C4—N1	118.39 (11)	C24—C25—N4	119.60 (10)
C5—C4—N1	121.97 (10)	C26—C25—N4	120.99 (10)
C4—C5—H5	119.9	C25—C26—H26	119.7
C6—C5—C4	120.10 (10)	C27—C26—C25	120.52 (11)
C6—C5—H5	119.9	C27—C26—H26	119.7
C1—C6—C5	119.40 (11)	C22—C27—C26	119.38 (11)
C1—C6—H6	120.3	C22—C27—H27	120.3
C5—C6—H6	120.3	C26—C27—H27	120.3
C9—C7—C8	117.79 (9)	C30—C28—C29	116.83 (9)
N1—C7—C8	126.31 (10)	N4—C28—C29	126.40 (10)
N1—C7—C9	115.90 (10)	N4—C28—C30	116.77 (10)
C7—C8—H8A	109.5	C28—C29—H29A	109.5
C7—C8—H8B	109.5	C28—C29—H29B	109.5
C7—C8—H8C	109.5	C28—C29—H29C	109.5
H8A—C8—H8B	109.5	H29A—C29—H29B	109.5
H8A—C8—H8C	109.5	H29A—C29—H29C	109.5
H8B—C8—H8C	109.5	H29B—C29—H29C	109.5
C10—C9—C7	120.17 (9)	C31—C30—C28	120.98 (9)
N2—C9—C7	116.86 (9)	N5—C30—C28	116.05 (9)
N2—C9—C10	122.96 (10)	N5—C30—C31	122.91 (10)
C9—C10—H10	120.7	C30—C31—H31	120.8
C11—C10—C9	118.57 (10)	C32—C31—C30	118.31 (10)
C11—C10—H10	120.7	C32—C31—H31	120.8
C10—C11—H11	120.5	C31—C32—H32	120.3
C10—C11—C12	119.05 (11)	C33—C32—C31	119.35 (10)
C12—C11—H11	120.5	C33—C32—H32	120.3
C11—C12—H12	120.7	C32—C33—H33	120.7
C11—C12—C13	118.68 (10)	C32—C33—C34	118.54 (10)
C13—C12—H12	120.7	C34—C33—H33	120.7
C12—C13—C14	120.18 (10)	C33—C34—C35	120.98 (10)
N2—C13—C12	122.87 (10)	N5—C34—C33	122.76 (10)
N2—C13—C14	116.92 (9)	N5—C34—C35	116.20 (10)
C15—C14—C13	118.42 (10)	C34—C35—C36	116.71 (10)
N3—C14—C13	116.05 (10)	N6—C35—C34	116.71 (10)
N3—C14—C15	125.49 (10)	N6—C35—C36	126.43 (11)
C14—C15—H15A	109.5	C35—C36—H36A	109.5
C14—C15—H15B	109.5	C35—C36—H36B	109.5
C14—C15—H15C	109.5	C35—C36—H36C	109.5
H15A—C15—H15B	109.5	H36A—C36—H36B	109.5
H15A—C15—H15C	109.5	H36A—C36—H36C	109.5
H15B—C15—H15C	109.5	H36B—C36—H36C	109.5
C17—C16—N3	119.44 (10)	C38—C37—N6	116.87 (11)
C21—C16—C17	119.39 (10)	C42—C37—C38	118.98 (11)
C21—C16—N3	120.71 (11)	C42—C37—N6	123.86 (11)
C16—C17—H17	119.8	C37—C38—H38	119.5
C18—C17—C16	120.49 (11)	C39—C38—C37	120.95 (12)
C18—C17—H17	119.8	C39—C38—H38	119.5
C17—C18—H18	120.5	C38—C39—H39	120.5
C19—C18—C17	119.08 (11)	C40—C39—C38	119.09 (12)
C19—C18—H18	120.5	C40—C39—H39	120.5
C18—C19—Cl2	119.41 (9)	C39—C40—C41	121.24 (11)
C20—C19—C18	121.38 (10)	C39—C40—Cl4	119.16 (10)
C20—C19—Cl2	119.21 (9)	C41—C40—Cl4	119.58 (10)
C19—C20—H20	120.5	C40—C41—H41	120.3
C19—C20—C21	119.10 (11)	C40—C41—C42	119.41 (12)
C21—C20—H20	120.5	C42—C41—H41	120.3
C16—C21—C20	120.49 (11)	C37—C42—C41	120.32 (12)
C16—C21—H21	119.8	C37—C42—H42	119.8
C20—C21—H21	119.8	C41—C42—H42	119.8
C7—N1—C4	121.10 (10)	C28—N4—C25	120.59 (9)
C9—N2—C13	117.87 (9)	C30—N5—C34	118.12 (9)
C14—N3—C16	122.28 (10)	C35—N6—C37	122.78 (10)

### Hydrogen-bond geometry (Å, º)

Cg1, Cg2, Cg4, Cg5 and Cg6 are the centroids of rings N2/C9–C13, C1–C6, N5/C30–C34, C22–C27 and C37–C42, respectively.

**Table:**

*D*—H···*A*	*D*—H	H···*A*	*D*···*A*	*D*—H···*A*
C20—H20···*Cg*6	0.95	2.94	3.6735 (14)	135
C32—H32···*Cg*1	0.95	2.73	3.3273 (12)	121
C2—H2···*Cg*4^i^	0.95	2.67	3.4012 (13)	134
C10—H10···*Cg*5^ii^	0.95	2.81	3.6446 (13)	147
C17—H17···*Cg*1^iii^	0.95	2.70	3.5850 (14)	155
C31—H31···*Cg*2^ii^	0.95	2.93	3.5795 (12)	127

Symmetry codes: (i) *x*, *y*+1, *z*; (ii) −*x*+1, −*y*+1, −*z*+1; (iii) −*x*+1, −*y*+1, −*z*.
